# Technical feasibility and short-term outcomes of laparoscopic isolated caudate lobe resection: an IgoMILS (Italian Group of Minimally Invasive Liver Surgery) registry-based study

**DOI:** 10.1007/s00464-021-08434-w

**Published:** 2021-03-31

**Authors:** Andrea Ruzzenente, Andrea Ciangherotti, Luca Aldrighetti, Giuseppe Maria Ettorre, Luciano De Carlis, Alessandro Ferrero, Raffaele Dalla Valle, Giuseppe Tisone, Alfredo Guglielmi, The IgoMILS – Sg1 Group, Francesca Ratti, Enrico Gringeri, Nadia Russolillo, Tommaso Campagnaro, Simone Conci, Giovanni Battista. Levi Sandri, Francesco Ardito, Ugo Boggi, Salvatore Gruttadauria, Luca Viganò, Fabrizio Di Benedetto, Giogio Ettore. Rossi, Stefano Berti, Graziano Ceccarelli, Leonardo Vincenti, Umberto Cillo, Felice Giuliante, Vincenzo Mazzaferro, Elio Jovine, Fulvio Calise, Giulio Belli, Fausto Zamboni, Andrea Coratti, Pietro Mezzatesta, Roberto Santambrogio, Giuseppe Navarra, Antonio Giuliani, Fabio Ferla, Antonio Domenico. Pinna, Amilcare Parisi, Michele Colledan, Abdallah Slim, Adelmo Antonucci, Gian Luca Grazi, Antonio Frena, Giovanni Sgroi, Alberto Brolese, Luca Morelli, Antonio Floridi, Alberto Patriti, Luigi Veneroni, Luigi Boni, Piero Maida, Guido Griseri, Marco Filauro, Silvio Guerriero, Raffaele Romito, Umberto Tedeschi, Giuseppe Zimmitti

**Affiliations:** 1 Department of Hepatobiliary Surgery, Unit of Hepato-Pancreato-Biliary Surgery, G. B. Rossi Hospital, University of Verona Medical School, Verona, Italy; 2 Hepatobiliary Surgery, IRCCS San Raffaele Hospital, Milan, Italy; 3 Division of General Surgery and Liver Transplantation, S. Camillo Hospital, Rome, Italy; 4 Surgical and Transplant Department, ASST Grande Ospedale Metropolitano Niguarda, Milan, Italy; 5 University of Milano-Bicocca School of Medicine, Milan, Italy; 6 Department of HPB and Digestive Surgery, Ospedale Mauriziano Umberto I, Turin, Italy; 7 Department of Surgery, Parma University Hospital, Parma, Italy; 8 Department of Transplant Surgery, Tor Vergata Foundation, Tor Vergata University, Rome, Italy

**Keywords:** Caudate lobe, Minimally invasive liver surgery, Multi-institutional, Propensity score matched

## Abstract

**Background:**

Although isolated caudate lobe (CL) liver resection is not a contraindication for minimally invasive liver surgery (MILS), feasibility and safety of the procedure are still poorly investigated. To address this gap, we evaluate data on the Italian prospective maintained database on laparoscopic liver surgery (IgoMILS) and compare outcomes between MILS and open group.

**Methods:**

Perioperative data of patients with malignancies, as colorectal liver metastases (CRLM), hepatocellular carcinoma (HCC), intrahepatic cholangiocarcinoma (ICC), non-colorectal liver metastases (NCRLM) and benign liver disease, were retrospectively analyzed. A propensity score matching (PSM) analysis was performed to balance the potential selection bias for MILS and open group.

**Results:**

A total of 224 patients were included in the study, 47 and 177 patients underwent MILS and open isolated CL resection, respectively. The overall complication rate was comparable between the two groups; however, severe complication rate (Dindo–Clavien grade ≥ 3) was lower in the MILS group (0% versus 6.8%, *P* = ns). In-hospital mortality was 0% in both groups and mean hospital stay was significantly shorter in the MILS group (*P* = 0.01). After selection of 42 MILS and 43 open CL resections by PSM analysis, intraoperative and postoperative outcomes remained similar except for the hospital stay which was not significantly shorter in MILS group.

**Conclusions:**

This multi-institutional cohort study shows that MILS CL resection is feasible and safe. The surgical procedure can be technically demanding compared to open resection, whereas good perioperative outcomes can be achieved in highly selected patients.

Isolated caudate lobe (CL) resection, originally described in 1990 by Lerut et al. [1], is an uncommon and technically challenging liver surgery procedure. Conversely, the association of CL resection and major hepatectomy (i.e., left or right hepatectomy) is more frequently performed.

CL is an autonomous segment which is located anterior to the inferior vena cava (IVC) and posterior to the liver hilum. Therefore, the surgical approach of this area can be technically difficult.

This segment can be divided into three regions according to Kumon et al. [2] classification: the Spiegel’s lobe, the paracaval portion and the caudate process. The Spiegel lobe is located behind the lesser omentum, on the left side of the intrahepatic IVC. The paracaval portion is located ventrally to the hepatic IVC, between the Spiegel lobe and the right lobe and below the hepatic veins. The caudate process is a projection of the liver between the IVC and the right hepatic lobe, close to the paracaval portion [3].

Minimally invasive liver surgery (MILS) largely increased after the first international consensus conference in Louisville in 2008 [4, 5] and it is nowadays the standard procedure for anteroinferior segments. MILS for posterior and superior segments is technically more challenging and is reserved to experienced surgeons in tertiary centers [6].

The safety and feasibility of MILS have been widely described, demonstrating shorter hospital stay, lower blood loss with comparable oncological outcomes when compared with the open approach.

MILS approaches to the CL have specific technical challenges: proximity to the IVC, major hepatic veins and the hepatic hilum [7]. Surgical series of MILS for the caudate lobe in the literature are limited and only a few reports describing more than 5 cases have been published [5, 8–16].

The aim of our study was to evaluate the safety and feasibility of MILS for caudate lobe resections in the Italian prospective maintained database on laparoscopic liver surgery (IgoMILS) through comparison with a cohort of patients submitted to open CL resection.

## Patients and methods

Patients who underwent MILS CL resection from March 2015 to December 2018 were identified from the IgoMILS registry, a prospective multi-institutional Italian database of patients undergoing laparoscopic liver resections. Specifically, data from 16 centers that performed at least 1 laparoscopic isolated CL resection were collected. Each center performed a variable number (1–13 cases) of MILS CL resections. Characteristics of the IgoMILS registry have been published previously [17]. In short, it is a prospective intention-to-treat registry open to any Italian center performing MILS, without restriction criteria based on number of procedures performed. The registry has been developed through eClinical, an electronic platform for the management of clinical trials. Any center willing to register patients in the registry was given access through the web page, https://www.cr-technology.com/igomils/.

Encryption data for identification of patients were held by the centers. Ethical approval for the registry was granted by the Ethics Committee of San Raffaele Hospital, Milan. The informed consent and study documents were evaluated and approved by the center where the subject was enrolled. Investigators belonging to the IgoMILS association undertake data auditing periodically. An external audit was performed in April 2018 to check the quality and reliability of the data. A random sample of 10% of patients enrolled in each center was selected. The registered data for each patient were verified; data accuracy was 96% and was similar in all centers.

Perioperative data of patients who underwent open isolated CL resection from November 2000 to December 2018 in the same surgical centers of the IgoMILS registry were also included in the study.

Our study population included both patients with malignancies, such as colorectal liver metastases (CRLM), hepatocellular carcinoma (HCC), intrahepatic cholangiocarcinoma (ICC), non-colorectal liver metastases (NCRLM) and benign liver disease.

The following preoperative data were collected for each patient: demographics, comorbidities, liver parenchyma characteristics, indication for resection and previous surgery. Operative details included the type of caudate lobe liver resection, the rate of conversion to open surgery, the use and type of pedicle clamping, surgical radicality, associated resections (hepatic or of other organs), intraoperative blood transfusions and blood losses. Early results included overall and severe complications, in-hospital mortality, postoperative blood transfusions, and length of stay. Pathological reports were available for all specimens. R0 resection was defined as a tumor-free surgical margin greater than 1 mm.

Caudate lobe anatomy was defined according to Kumon’s classification; for data collection and analysis we defined two different types of caudate lobe resection: isolated Spiegel’s lobe resection (S1-S) or combined resection of Spiegel’s lobe with paracaval portion and complete/partial resection of caudate process (S1-S + P).

Patients who underwent major liver resection with en-bloc CL resection or CL resection en-bloc with other hepatectomies were excluded from the study.

**Fig. 1:**
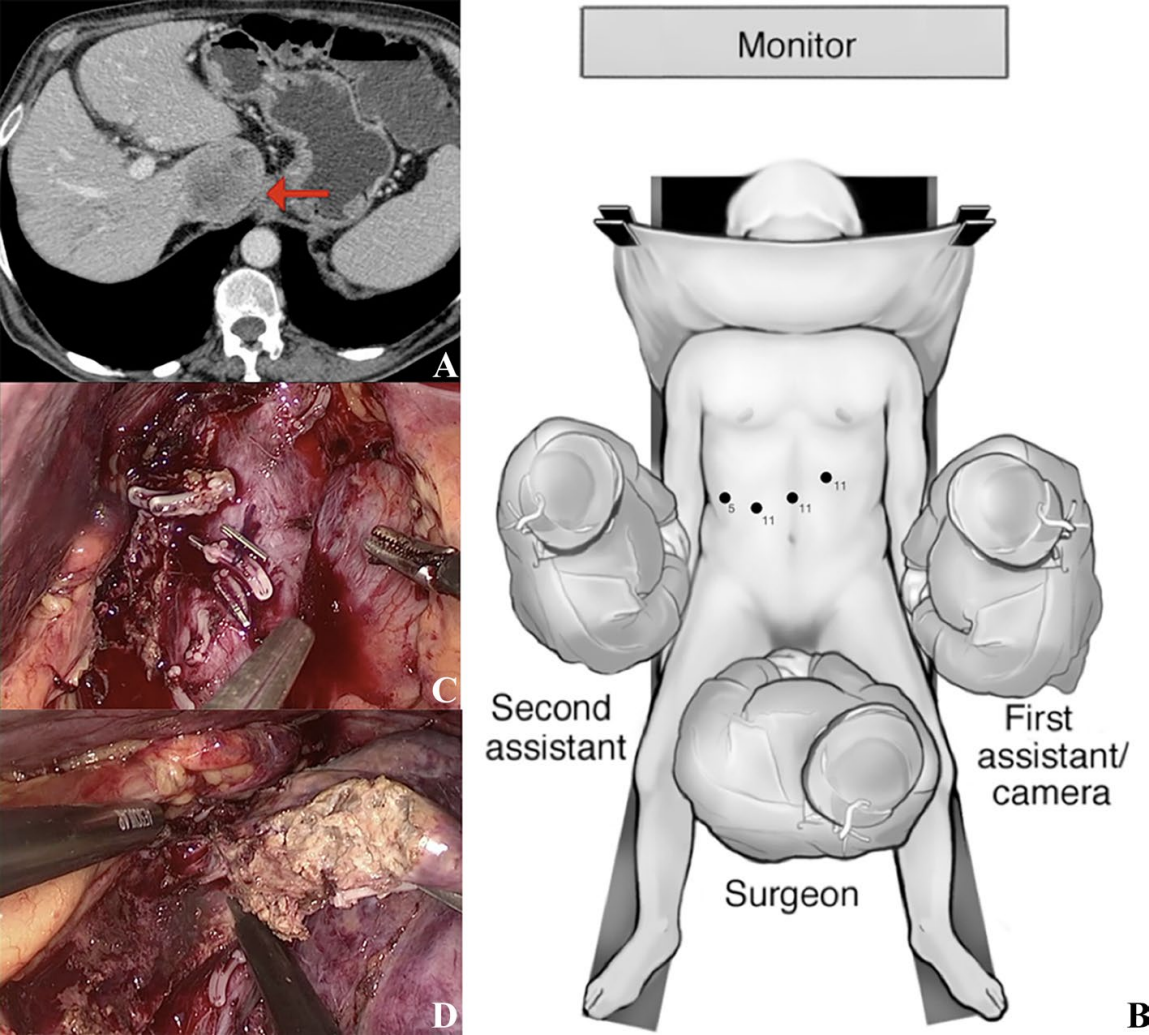
A surgical case of isolated MILS caudate lobe resection for HCC. Surgical case of isolated caudate lobe resection for 3 cm HCC (**A**), patient was positioned in French position with operating surgeon standing between patients’ legs, the scheme illustrating the position of the of trocars (**B**). Mobilization of caudate lobe from inferior vena cava, short hepatic veins secured with clips (**C**). Liver transection with ultrasonic aspirator (**D**)

Figure 1 illustrates a surgical case of isolated MILS caudate lobe resection for HCC.

Morbidity included all postoperative complications and was graded according to the Clavien–Dindo classification, and complications of grade 3 or higher were defined as severe complications [18]. Cumulative morbidity was measured using the Comprehensive Complication Index (CCI) [19].

## Statistical analysis

Categorical variables were compared using the *χ*^2^ test, Fisher’s exact test or Pearson’s test, as appropriate. The Student *t* test or 1-way ANOVA was used for continuous variables where appropriate. The results were expressed as mean and standard deviation (SD) or median and range.

To account for potential residual confounders regarding the effect of surgical approach on outcomes, propensity scores were estimated using a logistic regression model with the type of surgical approach as a dependent variable specified as MILS versus Open approach. Tumor size, liver histology, type of surgical procedure and tumor histology were independent variables in the logistic regression model. An exact propensity-score value was used for matching. The degrees of covariate imbalance were measured using the standardized (mean and proportion) differences, as proposed by Austin et al. [20]. A *P *value of < 0.05 (two-tailed) was considered statistically significant. All statistical analyses were performed with SPSS® version 25 (IBM) and XLSTAT (Addinsoft).

## Results

A total of 224 patients were included in the study, 47 (21%) patients underwent MILS and 177 (79%) patients underwent open approach.

The two groups were similar for demographic characteristics, but HBV infection rate was higher in the MILS group than in the open group (*P* = 0.034). Moreover, liver cirrhosis and fibrosis were more frequent in the MILS group than in the open group (*P* < 0.001). Regarding histological diagnosis, the frequency of benign disease was similar between the two groups. On the contrary, in the open group more CRLM were observed and in the MILS group more HCC were observed (*P* = 0.020). Demographic and preoperative characteristics of the two groups are reported in Table 1.

**Table 1:** Demographics and preoperative data

Variables	Before propensity matching score			After propensity matching score		
	S1-MILS	S1-open	*P* value	S1-MILS	S1-open	*P* value
*N*	47	177		42	43	
Gender male (%)	24 (51.1)	109 (61.6)	n.s	20 (47.6)	24 (55.8)	n.s
Age, mean, years (SD)	60.7 (15.7)	58.4 (14.5)	n.s	60.8 (15.9)	54.9 (15.7)	n.s
BMI, mean (SD)	25.4 (4.1)	25.3 (4.0)	n.s	25.3 (4.2)	24.9 (4.2)	n.s
Morbidities						
Arterial hypertension (%)	16 (35.6)	48 (28.7)	n.s	15 (36.6)	7 (16.3)	0.03
COPD (%)	3 (6.8)	9 (5.4)	n.s	3 (7.3)	3 (7.0)	n.s
Diabetes (%)	5 (11.4)	23 (13.9)	n.s	5 (12.2)	5 (11.6)	n.s
Dyslipidemia (%)	3 (6.8)	23 (13.8)	n.s	3 (7.3)	7 (16.3)	n.s
Ischemic heart disease (%)	3 (6.8)	14 (8.4)	n.s	3 (7.3)	5 (11.6)	n.s
HBV infection (%)	6 (13.6)	7 (4.2)	0.034	5 (11.6)	5 (12.2)	n.s
HCV infection (%)	7 (16.3)	11 (6.7)	n.s	7 (17.5)	8 (18.6)	n.s
Alcohol related liver dis. (%)	3 (6.8)	9 (5.5)	n.s	3 (7.3)	2 (4.7)	n.s
Previous chemotherapy (%)	16 (36.4)	49 (30.6)	n.s	15 (36.6)	8 (18.6)	n.s
Liver disease			< 0.001			n.s
Normal (%)	24 (51.1)	88 (49.7)		21 (50.0)	22 (51.2)	
Steatosis (%)	6 (12.8)	26 (14.7)		6 (14.0)	6 (14.3)	
Fibrosis (%)	6 (12.8)	5 (2.8)		5 (11.9)	4 (9.3)	
Cirrhosis (%)	10 (21.3)	16 (9.0)		10 (23.8)	11 (25.6)	
Missing (%)	1 (2.1)	42 (23.7)		0 (0.0)	0 (0.0)	
Pathology			0.020			n.s
CRLM (%)	12 (25.5)	87 (49.2)		11 (26.2)	13 (30.2)	
ICC (%)	0 (0.0)	5 (2.9)		0 (0.0)	1 (2.3)	
HCC (%)	17 (36.2)	29 (16.4)		16 (38.1)	13 (30.2)	
NCRLM (%)	4 (8.5)	15 (8.5)		3 (7.1)	3 (7.0)	
Benign (%)	13 (27.7)	39 (22.2)		12 (28.6)	13 (30.2)	
Missing (%)	1 (2.1)	2 (1.1)		0	0	
Previous surgery						
Cholecystectomy (%)	4 (8.5)	19 (13.5)	n.s	3 (7.1)	4 (9.3)	n.s
Hepatic resection (%)	5 (10.6)	27 (15.3)	n.s	4 (9.5)	4 (9.3)	n.s
Colonic resection (%)	9 (19.1)	44 (31.4)	n.s	9 (21.4)	11 (25.6)	n.s
Tumor size, mean, mm (SD)	36.9 (29.7)	31.1 (20.1)	n.s	35.7 (29.7)	37.7 (21.9)	n.s

Limited Spiegel’s lobe resections in the MILS and open group were 63.8% (30 patients) and 48.6% (86 patients), respectively. Instead, resection rate of Spiegel’s lobe extended at the paracaval portion and complete/partial resections of caudate process were 31.9% (15 patients) in the MILS group and 51.4% (85 patients) in the open group.

Pringle maneuver was performed in 29.8% (*n* = 14) of patients in the MILS group and 67.0% (*n* = 118) of patients in the open group (*P* < 0.001). Of note, the conversion rate from MILS approach to open surgery was 6.4% (*n* = 3) because of bleeding (1 case), intraperitoneal adhesions (1 case) and oncological radicality (1 case).

Mean intraoperative blood loss was lower in the MILS group compared to the open group (*P* < 0.001). No case of the MILS group required intraoperative blood transfusions, while 17 (10.0%) patients in the open group were transfused intraoperatively (*P* = 0.013). The use of abdominal drainage was less frequent in the MILS group than in the open group (*P* < 0.001).

Mean operation time in the MILS and open group was 309 min and 235 min, respectively (*P* = 0.001).

MILS and open group had similar mean size of the lesions and rate of positive resection margins for malignant tumors.

Overall complication rate was comparable between the two groups, but severe complication rate (Dindo–Clavien grade ≥ 3) was higher in the open group (6.8%, *n* = 12) compared to the MILS group (0%). Nevertheless, this difference was not statistically significant. On the contrary, mean CCI was 13.7 (SD ± 6.1) in the MILS group and 23.4 (SD ± 11.4) in the open group (*P* = 0.03). MILS group had a significantly shorter hospital stay (*P* = 0.01) and there were no in-hospital mortality in both groups. Intraoperative and postoperative data of both groups are summarized in Table 2.

**Table 2:** Intraoperative and postoperative data

Variables	Before propensity matching score			After propensity matching score		
	S1-MILS	S1-open	*p* value	S1-MILS	S1-open	*p* value
*N*	47	177		42	43	
Type of surgical procedure			0.022			n.s
Combined Spiegel’s lobe + paracaval portion (%)	15 (31.9)	91 (51.4)		13 (31.0)	16 (37.2)	
Limited Spiegel’s lobe (%)	30 (63.8)	86 (48.6)		29 (69.0)	27 (62.8)	
Missing (%)	2 (4.3)	0		0	0	
Associate surgical procedure						
Other liver resection (%)	13 (27.6)	51 (28.7)	n.s	11 (26.2)	5 (11.6)	n.s
Cholecystectomy (%)	6 (13.3)	25 (14.1)	n.s	6 (14.3)	7 (16.3)	n.s
Colonic resection (%)	3 (6.7)	9 (5.1)	n.s	3 (7.1)	1 (2.3)	n.s
Pringle maneuver			< 0.001			< 0.001
No (%)	31 (66.6)	31 (17.6)		26 (61.9)	10 (23.3)	
Intermittent (%)	14 (29.8)	118 (67.0)		14 (30.4)	23 (61.9)	
Continuous (%)	0	2 (1.1)		0 (0.0)	1 (2.3)	
Missing (%)	2 (4.3)	25 (14.2)		2 (4.8)	0	
Intraoperative data						
Conversion to open (%)	3 (6.4)	–		3 (7.1)	–	
Blood losses, mean, mL (SD)	175 (153)	343 (292)	< 0.001	173 (154)	290 (186)	0.003
Blood transfusions (%)	0 (0.0%)	17 (10.0)	0.013	0 (0.0)	2 (4.8)	n.s
Drainage (%)	35 (74.5)	173 (97.7)	< 0.001	30 (71.4%)	42 (97.7)	0.001
Operation time mean, min (SD)	309 (116)	235 (120)	0.001	298 (126)	238 (123)	0.038
Postoperative data						
R1 resection^a^ (%)	4/33 (12.1)	14/128 (10.9)	n.s	4/30 (13.3)	3/30 (10.0)	n.s
Blood transfusions (%)	3 (6.4)	12 (8.6)	n.s	3 (7.1)	6 (15.8)	n.s
In-hospital Mortality (%)	0	0	n.s	0	0	n.s
Overall complications (%)	8 (17.0)	34 (19.9)	n.s	6 (14.3)	3 (7.0)	n.s
Ascites (%)	0	3 (1.7)	n.s	0	0	n.s
Abdominal collection (%)	1 (2.1)	8 (4.7)	n.s	1 (2.4)	2 (4.7)	n.s
Biliary fistula (%)	0	7 (4.1)	n.s	0	1 (2.6)	n.s
Cardiological (%)	1 (2.1)	2 (1.4)	n.s	1 (2.4)	1 (2.6)	n.s
Bowel complications (%)	1 (2.1)	3 (2.1)	n.s	0	0	n.s
Pneumoniae (%)	1 (2.1)	3 (2.1)	n.s	0	0	n.s
Pleural effusions (%)	1 (2.1)	9 (5.2)	n.s	1 (2.4)	0	n.s
Wound infections (%)	1 (2.1)	2 (1.4)	n.s	0	0	n.s
Severe complications (Dindo–Clavien ≥ 3) (%)	0	12 (6.8)	n.s	0	3 (7.0)	n.s
CCI, mean (SD)	13.7 (6.1)	23.4 (11.7)	0.03	13.3 (6.0)	29.8 (6.3)	0.006
Length of stay, mean, days (SD)	4.9 (3.7)	8.7 (9.9)	0.01	4.7 (2.2)	7.4 (9.2)	n.s

^a^Referred only to malign tumors

To balance the potential selection bias, a PSM analysis was performed selecting 42 patients in the MILS and 43 patients in the open group. After the PSM analysis, demographics and preoperative data did not show significant differences between the two groups except for arterial hypertension that was more frequent in the MILS group (*P* = 0.03) (Table 1).

Most of the intraoperative and postoperative data also did not show significant differences (Table 2). However, MILS compared to open group had lower mean intraoperative blood losses [173 mL (SD ± 154 mL) versus 290 mL (SD ± 186 mL), *P* = 0.003], reduced use of abdominal drainage (71.4% vs 97.7%, *P* = 0.001), longer mean operation time (298 min vs 238 min, *P* = 0.038) and lower CCI [13.3 (SD ± 6.0) vs 19.8 (SD ± 6.3), *P* = 0.006].

### Subgroup analysis according to S1 type of resection

A subgroup analysis based on the type of surgical procedure in the MILS and open group was performed. In particular, we analyzed perioperative data of resections limited to Spiegel lobe (S1-S) and extended to the paracaval portion and complete/partial resection of caudate process (S1-S + P). Intraoperative and postoperative data of the subgroup analysis are reported in Table 3.

**Table 3:** Subgroup analysis according to the surgical procedure of S1 resection, resection limited to Spiegel lobe (S1-S) and resection of the Spiegel lobe extended to the paracaval portion and complete/partial resection of caudate process (S1-S + P)

	Limited Spiegel’s lobe (S1-S)			Spiegel’s lobe and paracaval portion (S1-S + P)			
	S1-MILS	S1-open	*P* value	S1-MILS		S1-open	*P* value
*N*	30	86		15	91		
Pringle maneuver			0.006				n.s
No (%)	20 (66.7)	15 (17.6)		8 (53.3)	16 (18.0)		
Intermittent (%)	10 (33.3)	64 (75.3)		5 (33.3)	54 (60.7)		
Continuous (%)	0	0		0	2 (2.2)		
Missing (%)	0	6 (7.1)		2 (13.3)	17 (19.1)		
Intraoperative data							
Conversion to open (%)	2 (6.7)	0	n.s	1 (6.7)	0		n.s
Blood losses, mean, mL (SD)	197.7 (158.1)	346.2 (307.8)	0.014	234 (127.4)	340 (277.2)		n.s
Drainage (%)	20 (66.7)	83 (97.6)	< 0.001	13 (86.7)	86 (97.7)		n.s
Operation time mean, min (SD)	294 (110)	240 (127)	0.044	327 (125)	216 (110)		0.003
Postoperative data							
R1 resection^a^ (%)	2/20 (10.0)	5/69 (7.2)	n.s	2/11 (18.2)	9/64 (14.1)		n.s
Tumor size, mean, mm (SD)	37.1 (32.3)	38.2 (87.1)	n.s	35.9 (25.0)	33.9 (20.5)		n.s
Blood transfusions (%)	2 (6.7)	7 (10.8)	n.s	1 (6.7)	5 (6.7)		n.s
In-hospital mortality (%)	0	0		0	0		
Overall complications (%)	5 (16.7)	13 (15.7)	n.s	3 (16.7)	21 (15.7)		n.s
Ascites (%)	0	2 (2.4)	n.s	0	1 (1.1)		n.s
Abdominal collection (%)	1 (3.3)	0	n.s	0	6 (6.8)		n.s
Biliary fistula (%)	0	2 (2.4)	n.s	0	5 (5.7)		n.s
Cardiological (%)	1 (3.3)	2 (2.4)	n.s	0	2 (2.4)		n.s
Bowel complications (%)	0	2 (2.4)	n.s	1 (6.7)	1 (1.3)		n.s
Pneumoniae (%)	0	1 (6.7)	n.s	1 (6.7)	3 (3.9)		n.s
Pleural effusions (%)	1 (3.3)	2 (2.4)	n.s		7 (8.0)		n.s
Wound infections (%)	0	1 (1.5)	n.s	1 (6.7)	1 (1.3)		n.s
Severe complications (Dindo Clavien ≥ 3) (%)	0	4 (4.7)	n.s	0	8 (9.0)		n.s
CCI, mean (SD)	11.8 (5.3)	22.2 (11.2)	n.s	12.7 (7.1)	24.2 (12.3)		n.s
Length of stay, mean days (SD)	4.63 (2.2)	8.0 (9.8)	n.s	4.67 (2.3)	9.43 (10.1)		n.s

Intraoperative and postoperative data

^a^Referred only to malign tumors

Among the intraoperative variables, the MILS group showed lower blood loss compared to the open group for both S1-S (197.7 mL vs 346.2 mL, *P* = 0.014) and S1-S + P (134 mL vs 340 mL, *P* = n.s). Instead, mean operation time was significantly longer in the MILS group compared to the open group for S1-S (294 min vs 240 min, *P* = 0.044) and S1-S + P (327 min vs 216 min, *P* = 0.003). Other postoperative variables, such as rate and severity of surgical complications and length of hospital stay, were comparable between the MILS and open group in both S1-S and S1-S + P.

## Discussion

In the last decades, MILS steadily increased. The spreading of MILS approach is supported by the improvements of surgical techniques, the implementation of new surgical tools and the growing MILS hepatobiliary surgeon’s skills that contribute to face more complex MILS procedures [21].

CL resection is a minor but complex surgical intervention, even with an open approach, due to its location deep into the liver. Anatomically, caudate lobe can be classified into three different parts: the Spiegel’s lobe that is located on the left side of the IVC, the paracaval portion in front of the IVC and the caudate process on the right side of the IVC [2].

Although the Spigelian lobe is relatively easy to manage, the resection of the caudate process and paracaval portion may be very demanding due to the relationship with the hepato-caval confluence, the main biliary ducts and the main portal vein bifurcation. MILS CL resection is a double-edged sword; indeed, MILS could improve short-term outcomes decreasing postoperative pain and shortening in-hospital stay, but is also true that surgeon should be aware of the potential intraoperative life-threatening massive bleeding caused by injury of IVC [22, 23].

In our experience, the main advantages of MILS are the magnification of surgical field that is very helpful during the dissection of small CL collaterals coming from IVC and the caudal view of segment 1 [26, 27]. Moreover, the balance between intra-abdominal pressure and central venous pressure can reduce venous bleeding (Fig. 1).

From a technical point of view, three approaches to the caudate lobe have been described: from the left side, from the right side and trans-parenchymal [28, 29]. The most common approach for the Spiegel’s lobe is from the left side because it exposes the anterior surface of IVC. Right side approach is more commonly used for lesions of the paracaval portion or the caudate process. The trans-parenchymal approach is more suitable for larger lesions but is rarely applied for its complexity also in open approach [30].

The oncological safety of CL resection can be a matter of debate since the size and location of the tumor and its relationship with major hilar structures and IVC may affect the ability to achieve negative resection margins.

Our multi-institutional study confirmed the feasibility and safety of MILS CL resection and showed that MILS approach had lower intraoperative blood loss, lower severe postoperative complication rate and shorter hospital stay compare to open approach. Furthermore, the MILS and open group had similar good oncologic outcomes in terms of negative resection margin rate.

Our conversion rate of 6.4% (*n* = 3) is comparable to other series reported in the literature. The reasons for the conversion to open surgery were major bleeding, the need to achieve oncological radicality and strong adhesions. No massive intraoperative bleedings and in-hospital mortality were observed.

The complexity of the surgical procedures of CL resection varies depending on the portions to be resected. In particular, the resection of the paracaval portion and/or caudate process is more challenging than isolated Spiegel’s lobe resection. In order to clearly elucidate the short-term outcomes of open and laparoscopic surgery of S1-S and S1-S-P resection, we performed a subgroup analysis. Limited S1-S resection by MILS approach showed lower blood loss, but longer operative time compared to open approach. Instead, in more complex operation, as S1-S-P resection, we found that only mean operative time was significantly longer by MILS approach. Current literature is very limited about this topic. Wang et al. [24] reported that patients who underwent open caudate lobectomy for tumors located at the paracaval portion or the caudate process had longer operative time, vascular occlusion time, hospital stay and higher blood loss compared to Spiegel’s lobe resection. Conversely, Zhao et al. [25] described similar short-term outcomes in patients submitted to different types of robotic-assisted CL resections (Spiegel’s lobe, paracaval portion and/or caudate process).

Few articles concerning the laparoscopic approach to CL are published in literature. Moreover, the majority of these studies are limited to case reports or small surgical series [5, 8–16]. Table 4 provides a literature review and demonstrates that morbidity rate of MILS CL resections ranged from 0 to 33% with no postoperative mortality.

**Table 4:** Literature review

First author	Year	Country	Type	Sample size	Surgical technique	Type of lesions	Morbidity/mortality	Operation time
Koffron	2007	USA	Case series	7	MILS	5 benign, 2 malignant	NR/0	NR
Chen	2013	China	Case series	8	MILS	malignant	0/0	201–345
Araki	2016	USA	Case-match	15	MILS	13 benign, 2 malignant	6.6%/0	60–480
Oh	2016	Korea	Case series	6	MILS	malignant	0/0	168–615
Salloum	2016	France	Case series	5	MILS	4 benign, 1 malignant	0/0	50–700
Chai	2018	China	Case series	6	MILS	2 benign, 4 malignant	33.3%/0	173–300
Jin	2018	China	Case series	12	MILS	5 benign, 7 malignant	0/0	140.8 (Mean)
Xu	2020	China	Case-match + PSM	19 (MILS) vs 112 (open)	MILS vs open	11 benign, 8 malignant (MILS) vs 57 benign, 55 malignant (open)	11.1%/0 (MILS) vs 11.1%/0 (open)	128.5–219 vs 163.75–238
Cappelle	2020	Belgium, Norway, The Netherlands	Case series	32	MILS	5 benign, 27 malignant	6.3% (severe)/0	29–440
Current study	2020	Italy	Case-match + PSM	47 (MILS) vs 177 (open)	MILS vs open	13 benign, 34 malignant (MILS) vs 38 benign, 139 malignant (open)	14.3%/0 (MILS) vs 7.0%/0 (open)	309 (mean) 235 (mean) vs

Only a few studies aimed to assess robotic approach to CL. One series of 10 cases describes the safety and feasibility of the robotic approach emphasizing the importance of the fully wristed dexterity, ergonomics and the full 3D vision [31]. Nevertheless, the authors reported equivalent short-term results with the laparoscopic approach, but with lower costs and shorter operative time. In our study, only 1 patient was treated with robotic approach.

The only comparative study between MILS and open approach was presented by Xu et al.[9] and demonstrated the benefits of the laparoscopic technique in reducing blood loss and transfusion rate. The reported morbidity and severe complication rate was similar to our study. In addition, the authors demonstrated a shorter time for ambulation, oral intake, first flatus and drainage tube removal in the MILS group [32, 33].

The present study has several limitations that should be considered. Despite the IgoMILS registry enrolls patients prospectively from several Italian centers, this is a retrospective study with a limited number of patients in the MILS group. Besides, we included lesions with different histologies. The PSM analysis was performed to mitigate the risk of selection bias; however, further studies are expected. Finally, MILS has been recently developed and the experience with difficult liver segments is improving. Therefore, short- and long-term outcomes should be reassessed in the future.

## Conclusions

This multi-institutional cohort study shows that MILS CL resection is feasible and safe. The surgical procedure can be technically demanding compared to open resection, whereas good perioperative outcomes can be achieved in selected cases.
